# Estimation of Wheat Plant Density at Early Stages Using High Resolution Imagery

**DOI:** 10.3389/fpls.2017.00739

**Published:** 2017-05-16

**Authors:** Shouyang Liu, Fred Baret, Bruno Andrieu, Philippe Burger, Matthieu Hemmerlé

**Affiliations:** ^1^UMR EMMAH, INRA, UAPVAvignon, France; ^2^UMR ECOSYS, INRA, AgroParisTech, Université Paris-SaclayThiverval-Grignon, France; ^3^UMR AGIR, INRACastanet Tolosan, France; ^4^Hi-PhenAvignon, France

**Keywords:** plant density, RGB imagery, neural network, wheat, recursive feature elimination, Hough transform

## Abstract

Crop density is a key agronomical trait used to manage wheat crops and estimate yield. Visual counting of plants in the field is currently the most common method used. However, it is tedious and time consuming. The main objective of this work is to develop a machine vision based method to automate the density survey of wheat at early stages. RGB images taken with a high resolution RGB camera are classified to identify the green pixels corresponding to the plants. Crop rows are extracted and the connected components (objects) are identified. A neural network is then trained to estimate the number of plants in the objects using the object features. The method was evaluated over three experiments showing contrasted conditions with sowing densities ranging from 100 to 600 seeds⋅m^-2^. Results demonstrate that the density is accurately estimated with an average relative error of 12%. The pipeline developed here provides an efficient and accurate estimate of wheat plant density at early stages.

## Introduction

Wheat is one of the main crops cultivated around the world with sowing density usually ranging from 150 to 400 seed⋅m^-2^. Plant population density may significantly impact the competition among plants as well as with weeds and consequently affect the effective utilization of available resources including light, water, and nutrients (Shrestha and Steward, 2003; Olsen et al., 2006). Crop density appears therefore as one of the important variables that drive the potential yield. This explains why this information is often used for the management of cultural practices (Godwin and Miller, 2003). Plant population density is still investigated most of the time by visually counting the plants in the field over samples corresponding either to a quadrat or to a segment. This is achieved at the stage when the majority of plants have just emerged and before the beginning of tillering (Norman, 1995) which happens few days to few weeks after emergence. This method is time and labor intensive and may be prone to human error.

Some efforts have been dedicated to the development of high-throughput methods for quantifying plant density. This was mainly applied to maize using either capacitive sensors during the harvest (Nichols, 2000; Li et al., 2009) or optical sensors including 2D cameras (Shrestha and Steward, 2003, 2005; Tang and Tian, 2008a,b) and range sensors (Jin and Tang, 2009; Nakarmi and Tang, 2012, 2014; Shi et al., 2013, 2015). However, quantifying the population density of maize is much simpler than that of wheat since maize plants are normally bigger, with larger plant spacing along the row and more evenly distributed. In wheat crops, leaves between neighboring plants overlap rapidly, and tillers will also appear, making the plant identification very difficult when they have more than three leaves, even using visual counting in the field. Most studies during these early stages report results derived from estimates of the vegetation fraction coverage measured using high resolution imagery (Guo et al., 2013) or based on vegetation indices computed with either multispectral (Sankaran et al., 2015) or hyperspectral (Liu et al., 2008) reflectance measurements. However, none of these investigations specifically addressed the estimation of plant density. Advances in digital photography providing very high resolution images, combined with the development of computer vision systems, offer new opportunities to develop a non-destructive high-throughput method for plant density estimation.

The objective of this study is to develop a system based on high resolution imagery that measures wheat plant population density at early stages. The methods used to acquire the RGB images and the experimental conditions are first presented. Then the pipeline developed to process the images is described. Finally, the method is evaluated with emphasis on its accuracy and on its corresponding domain of validity.

## Materials and Methods

### Field Experiments and Measurements

Three experiments were conducted in 2014 in France (**Table 1**): Avignon, Toulouse, and Paris. In Avignon, four sowing densities (100, 200, 300, and 400 seeds⋅m^-2^) with the same “Apache” cultivar were sampled. In Toulouse, five plant densities (100, 200, 300, 400, and 600 seeds⋅m^-2^) with two different cultivars, “Apache” and “Caphorn” were considered. In Paris, two cultivars with a single sowing density of 150 seeds⋅m^-2^ were sampled. All measurements were taken around 1.5 Haun stage, when most plants already emerged. A total of 16 plots were therefore available over the three experiments under contrasted conditions in terms of soil, climate, cultivars, and sowing densities. All the plots were at least 10 m length by 2 m width.

**Table 1:** Characteristics of the three experimental sites.

Sites	Latitude	Longitude	Cultivars	Sowing density (seeds⋅m^-2^)	Reference density (plants⋅m^-2^)	Illumination conditions	Cameras	Resolution	Focal length	Spatial resolution (mm)
Toulouse	43.5°N	1.5°E	Apache	100, 200, 300, 400, 600	106, 187, 231, 350, 525	Diffuse	Sigma SD14	2640 by 1760	50 mm	0.23
			Caphorn	100, 200, 300, 400, 600	118, 206, 250, 387, 431					
Paris	48.8°N	1.9°E	Premio	150	154	Flash	NIKON D5200	4496 by 3000		0.16
			Attlass	150	182					
Avignon	43.9°N	4.8°E	Apache	100, 200, 300, 400	54, 129, 232, 425	Direct	Sigma SD14	4608 by 3072		0.13


**FIGURE 1:**
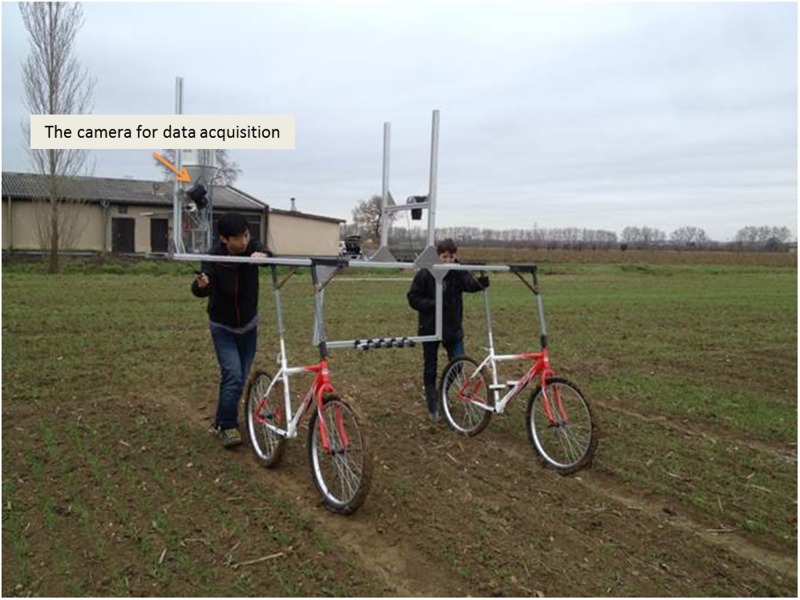
**The image acquisition in the field with the Phenotypette**.

In Toulouse and Avignon, images were acquired using an RGB camera fixed on a light moving platform, termed Phenotypette (**Figure 1**). The platform was driven manually at about 0.5 m⋅s^-1^. For each plot, at least 10 images were collected to be representative of the population. For Paris experiment, the camera was mounted on a monopod to take two pictures with no overlap. In all the cases, the camera was oriented at 45° inclination perpendicular to the row direction and was pointing at the center row from a distance of 1.5 m and with spatial resolution around 0.2 mm (**Figure 1** and **Table 1**). For each plot, 10 images were selected randomly among the whole set of images acquired. The number of plants located in the two central rows was then visually counted over each of the 10 selected images to derive the reference plant density.

### Image Processing

**FIGURE 2:**
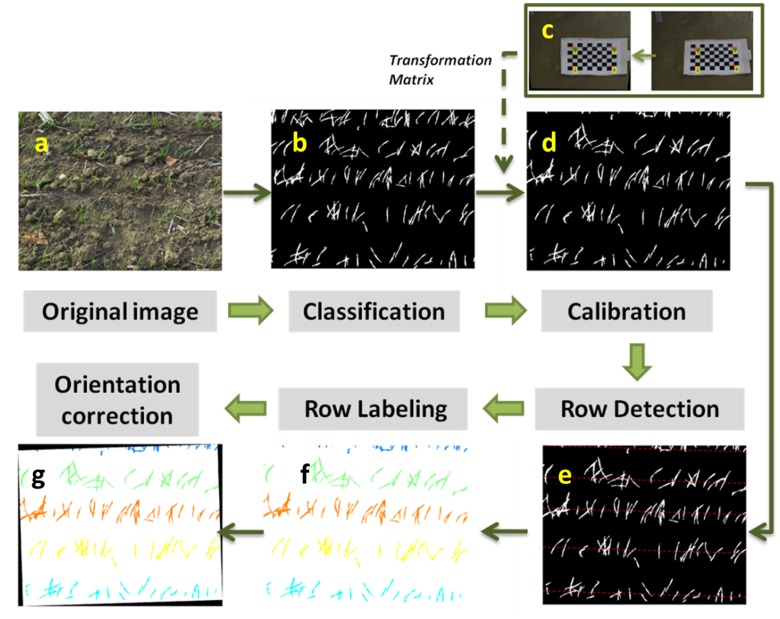
**The methodology involving image processing feature extraction.** **(a)** Original image. **(b)** Binary image. **(c)** Image of a chessboard to derive the transformation matrix. **(d)** Calibrated image. **(e)** Detecting rows in the image, corresponding to red dashed lines. **(f)** Labeling rows with different colors. **(g)** Correcting row orientation to be horizontal.

Each image was processed using the pipeline sketched on **Figure 2**. It was mainly programmed using MATLAB and Image Processing Toolbox R2016a (code available on request). To facilitate the application, the corresponding MATLAB functions used are also given in the text.

#### Classification of Green Elements

The images display green pixels corresponding to the emerged plants, and brown pixels corresponding to the soil background. The RGB color space was firstly transformed into Lab, to enhance its sensitivity to variations in greenness (Philipp and Rath, 2002). The Otsu automatic thresholding method (Otsu, 1975) was then applied to channel ‘a’ to separate the green from the background pixels (function: *graythresh*). Results show that the proposed method performs well (**Figure 2b**) under the contrasted illumination conditions experienced (**Table 1**). Further, this approach provides a better identification of the green pixels (results not presented for the sake of brevity) as compared to the use of supervised methods (Guo et al., 2013) based on indices such as the excess green (Woebbecke et al., 1995) or more sophisticated indices proposed by Meyer and Neto (2008).

#### Geometric Transformation

The perspective effect creates a variation of the spatial resolution within the image: objects close to the lens appear large while distant objects appear small. A transformation was therefore applied to remap the image into an orthoimage where the spatial resolution remains constant. The transformation matrix was calibrated using an image of a chessboard for each camera setup (**Figure 2c**). The chessboard covered the portion of the image that was later used for plant counting. The corners of the squares in the chessboard were identified automatically (function: *detectCheckerboardPoints*). Then the transformation matrix can be derived once the actual dimension of the squares of the chessboard is provided (function: *fitgeotrans*) (**Figure 2c**). The transformation matrix was finally applied to the whole image for given camera setup (function: *tformfwd*) (**Figure 2d**). This allows remapping the image into a homogeneously distributed domain on the soil surface.

#### Row Identification and Orientation

**FIGURE 3:**
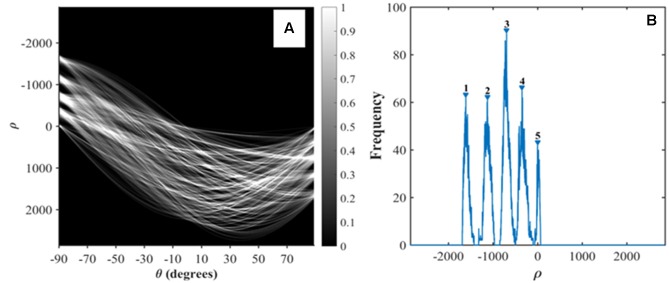
**Hough transform to detect rows.** **(A)** Hough transform. **(B)** Identification of the peaks of ρ corresponding to the rows.

The plant density measurement for row crops such as wheat is achieved by counting plants over a number of row segments of given length. Row identification is therefore a mandatory step as sketched in **Figure 2e**. Row identification methods have been explored intensively mostly for the automation of robot navigation in field (Vidović et al., 2016). Montalvo et al. (2012) reviewed the existing methods and found that the Hough transform (Slaughter et al., 2008) is one of the most common and reliable methods. It mainly involves computing the co-distribution of the length (ρ) and orientation (θ) of the segments defined by two green pixels (**Figure 3A**). The Hough transform detects dominant lines even in the presence of noise or discontinuous rows. The noise could include objects between rows such as weeds or misclassified background pixels such as stones (Marchant, 1996; Rovira-Más et al., 2005). Although the Hough transform is computationally demanding, its application on edge points of the green objects decreases this constraint. Hence, the ‘Canny Edge Detector’ (Canny, 1986) was consequently used to detect edges prior to the application of the Hough transform. The Hough transform was conducted with orientation -90°<θ<90° with 0.1° angular steps and a radius -3000 < ρ < 3000 pixels with 1 pixel steps (function: *hough*) (**Figure 3A**).

Five main components show up in the image (**Figure 3A**), corresponding to the five rows of the original image (**Figure 2a**). As all rows are expected to be roughly parallel, their orientation could be inferred as the θ value, θ_row_ (where θ_row_ = 90° corresponds to the horizontal orientation on the images on **Figure 2f**), that maximizes the variance of ρ. The positions of the rows are derived from the peaks of frequency for θ = θ_row_ (**Figure 3B**). Five lines on **Figure 2e** highlight the center of each row. Because of the uncertainty in the orientation of the camera along the row, the row line drawn on the images are not exactly horizontal. This is illustrated in **Figure 2f** where θ_row_ = - 88.2°. The images were therefore rotated according to θ_row_ (function: *imrotate*), so that the rows are strictly horizontal in the displayed images **Figure 2g**.

#### Object Identification and Feature Extraction

An object in a binary image refers to a set of pixels that form a connected group with the connectivity of eight neighbors. Each object was associated to the closest row line and characterized by 10 main features (function: *bwmorph*) (the top 10 features in **Table 2**). Three additional features were derived from skeletonization of the object: the length, number of branch and end points of the skeleton (function: *regionprops*) (the last three features in **Table 2**). More details on the feature extraction function used can be found in https://fr.mathworks.com/help/images/.

**Table 2:** The 13 features extracted for each of the connected object.

#	Name	Meaning	Unit
F1	Area	Number of pixels of the connected component (object)	Pixel
F2	FilledArea	Number of pixels of the object with all holes filled	Pixel
F3	ConvexArea	Number of pixels within the associated convex hull	Pixel
F4	Solidity	Ratio of number of pixels in the region to that of the convex hull	Scalar
F5	Extent	Ratio of number of pixels in the region to that of the bounding box	Scalar
F6	EquivDiameter	Diameter of a circle with the same area as the region	Pixel
F7	MajorAxisLength	Length of the major axis of the ellipse equivalent to the region.	Pixel
F8	MinorAxisLength	Length of the minor axis of the ellipse equivalent to the region.	Pixel
F9	Eccentricity	Eccentricity of the equivalent ellipse to the region	Scalar
F10	Orientation	Orientation of the major axis of the equivalent ellipse	Degree
F11	LengthSkelet	Number of pixels of the skeleton	Pixel
F12	NumEnd	Number of end points of the skeleton	Scalar
F13	NumBranch	Number of branch points of the skeleton	Scalar


### Estimation of the Number of Plants Contained in Each Object

Machine learning methods were used to estimate the number of plants contained in each object from the values of their 13 associated features (**Table 2**). Artificial neural networks (ANNs) have been recognized as one of the most versatile and powerful method to relate a set of variables to one or more variables. ANNs are interconnected neurons characterized by a transfer function. They combine the input values (the features of the object) to best match the output values (number of plants in our case) over a training database. The training process requires first to define the network architecture (the number of hidden layers and nodes per layer and the type of transfer function of each neuron). Then the synaptic weights and biases are tuned to get a good agreement between the number of plants per object estimated from the object’s features and the corresponding number of plants per object in the training database. A one-layer feed-forward network with *k_n_* tangent sigmoid hidden neurons and none linear neuron was used. The number of hidden nodes was varied between 1 ≤ *k_n_* ≤ 10 to select the best architecture. The weights and biases were initialized randomly. The training was achieved independently over each site considering 90% of the data set corresponding to a total of the 606 (Toulouse), 347 (Paris), and 476 (Avignon) objects. The remaining 10% objects of each site was used to evaluate the performance of the training. Note that the estimates of number of plants per object were continuous, i.e., representing actually the average probability of getting a discrete number of plants.

A compact, parsimonious and non-redundant subset of features should contribute to speed up the learning process and improve the generalization of predictive models (Tuv et al., 2009; Kuhn and Johnson, 2013; Louppe, 2014). Guyon et al. (2002) proposed recursive feature elimination (RFE) to select the optimal subtest of features. Specific to ANN, the combinations of the absolute values of the weights were used firstly to rank the importance of predictors (features) (Olden and Jackson, 2002; Gevrey et al., 2003). For the subset including *n* features, RFE presumes that the subset of the top *n* features outperforms the other possible combinations (Guyon et al., 2002; Granitto et al., 2006). Then 13 iterations corresponding to the 13 features need to be computed to select the optimal subset defined as the smallest set providing a RMSE_n_ lower than 1.02 RMSE_best_, where RMSE_best_ is the minimum RMSE value observed when using the 13 features. To minimize possible overfitting of the training dataset, a cross-validation scheme was used (Seni and Elder, 2010) with the training data set including 90% of the cases and the test data set containing the remaining 10%. The process was repeated five times with a random drawing of the training and test data sets for each trial.

## Results

### Number of Plants per Object and Object Feature Selection

**FIGURE 4:**
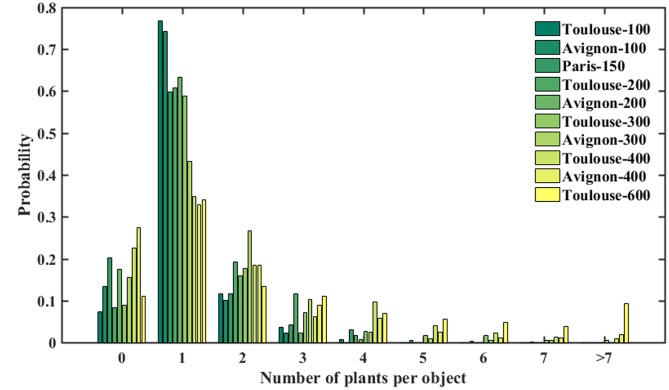
**Number of plants per object over the three sites**.

The number of plants per object resulted in a consistently right-skewed distribution over the three experimental sites (**Figure 4**). For all the plots, objects containing single plants have the highest probability of occurrence. However, objects contain generally more plants for high density as compared to the low density conditions. Note that 10–20% of the objects were classified as null, i.e., containing no plants. This corresponds to errors in separating plants from the background: objects such as straw residues, stone, or weeds may show colors difficult to separate in the classification step. Further, due to the variability of the illumination conditions, plants may be misclassified into two disconnected objects. In this case, the larger part is considered as a plant while the smaller remaining part is considered as non-plant, i.e., set to 0.

**FIGURE 5:**
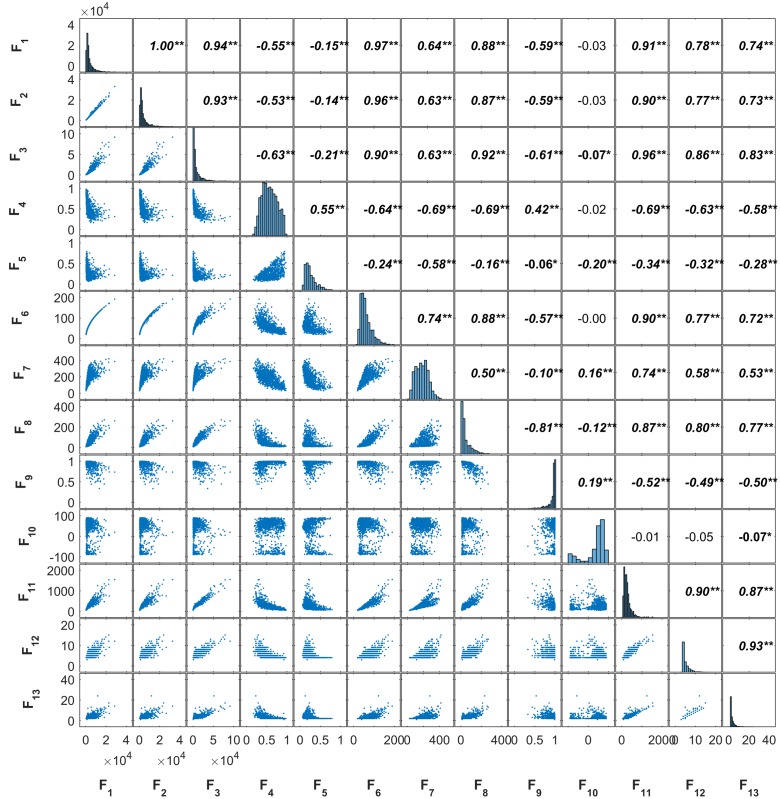
**The correlation among the 13 objects’ features for the Toulouse site (^∗∗^: 0.01, ^∗^: 0.05).** The abbreviation of features refers to names in **Table 2**.

Most of the 13 features described in **Table 2** are closely related as illustrated by the plot-matrix of the Toulouse site (**Figure 5**). Correlations are particularly high between the four area related features (F1, F2, F3, F6), between the skeleton derived features (F11, F12, F13), and between the area and skeleton related features. Similar correlations were observed over the Paris and Avignon sites. These strong relationships indicate the presence of redundancy between the 13 features, which may confuse the training of ANN. However, this could be partly overcome by the RFE feature selection algorithm.

**FIGURE 6:**
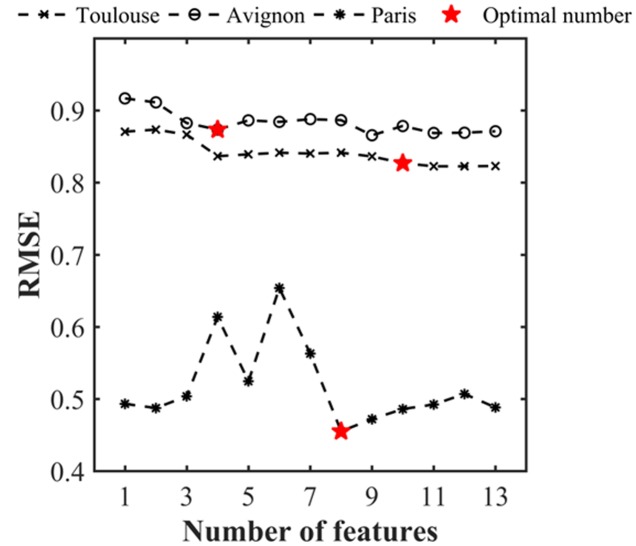
**RMSE associated to the estimates of the number of plants per object as a function of the number of features used.** The RMSE was evaluated over the test data set for each individual site.

The estimation performances of the number of plants per object were evaluated with the RMSE metrics as a function of the number of features used (**Figure 6** and **Table 3**). Note that the RMSE value was calculated based on the visual identification of the number of plants per object in the dataset. **Figure 6** shows that the RMSE decreases consistently when the number of features used increases. However, after using the first four features, the improvement in estimation performances is relatively small when including remaining features. The number of features required according to our criterion (1.02. RMSE_best_) varies from 10 (Toulouse) to 4 (Avignon). A more detailed inspection of the main features used across the three sites (**Table 4**) shows the importance of the area related features (F1, F2, F3, F4, and F6) despite their high inter-correlation (**Figure 5**). The length of the skeleton (F11) also appears important particularly for the Avignon site, while the orientation and extent do not help much (**Table 4**).

**Table 3:** Performance of the estimation of the number of plants per object over three experiments.

Sites	Training size	n_node_	Number of features	*R*^2^	RMSE	Bias
Toulouse	606	2	10	0.83	0.83	0.28
Paris	347	2	8	0.79	0.47	0.077
Avignon	476	2	4	0.61	0.87	0.45


**Table 4:** Features selected and the corresponding rank over three sites.

#	Features	Toulouse	Paris	Avignon
F_1_	Area	2	1	3
F_2_	FilledArea	1	3	
F_3_	ConvexArea	4	4	2
F_4_	Solidity	10	7	
F_5_	Extent			
F_6_	EquivDiameter	3	2	4
F_7_	MajorAxisLength			
F_8_	MinorAxisLength	5	5	
F_9_	Eccentricity	8	8	
F_10_	Orientation			
F_11_	LengthSkelet	6	6	1
F_12_	NumEnd	7		
F_13_	NumBranch	9		


**FIGURE 7:**
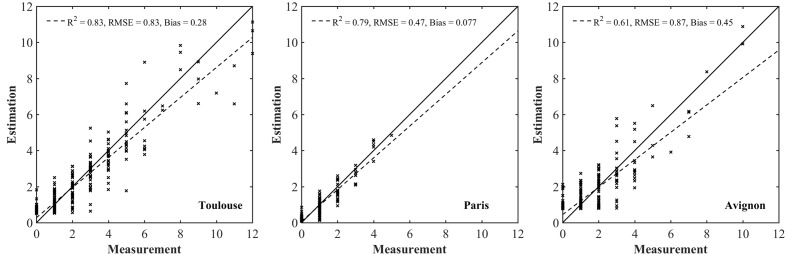
**Comparison between the estimated number of plants per object with the value measured over the test dataset for each individual site**.

As expected, the model performs the best for the Paris site (**Table 3**) where the situation is simpler because of the low density inducing limited overlap between plants (**Figure 4**). For sowing density <= 300 seeds⋅m^-2^, a better accuracy is reached in Toulouse (RMSE = 0.51) and Avignon (RMSE = 0.68) sites. Conversely, the larger number of null objects (**Figure 4**) corresponding to misclassified objects or split plants in the Avignon site, explains the degraded performance (**Table 3**). The bias in the estimation of the number of plants per object appears relatively small, except for the Avignon site. Attention should be paid on the bias since the application of the neural network on a larger number of objects is not likely to improve the estimation of the total number of plants. The bias is mostly due to difficulties associated to the misclassified objects (**Figure 7**). Note that the estimation performance degraded for the larger number of plants per objects (**Figure 7**) as a consequence of more ambiguities and smaller samples used in the training process.

### Performance of the Method for Plant Density Estimation

**FIGURE 8:**
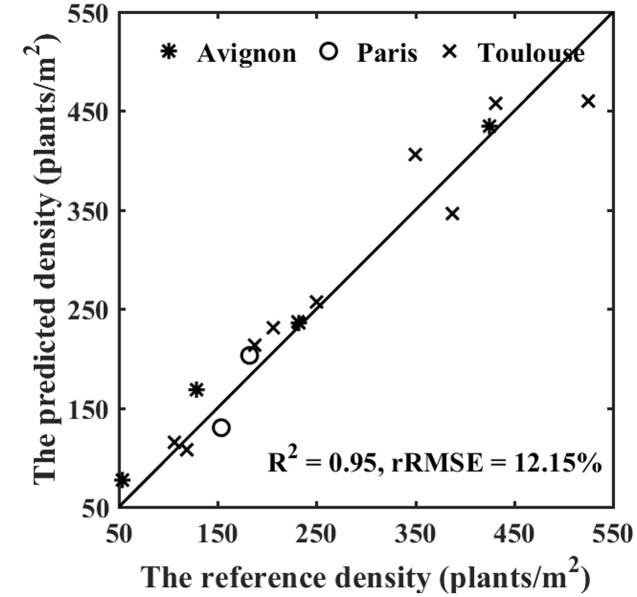
**Performance of density estimation over the three sites**.

The estimates of plant density were computed by summing the number of plants in all the objects extracted from the row segments identified in the images, divided by the segment area (product of the segment length and the row spacing). The reference density was computed from the visually identified plants. Results show a good agreement between observations and predictions over sowing densities ranging from 100 to 600 plants⋅m^-2^ (**Figure 8**). The performances slightly degrade for densities higher than 350 plants⋅m^-2^. This may be explained by the difficulty to handle more complex situation when plant spacing decreases, with a higher probability of plant overlap (**Figure 7**). Note that the slight overestimation observed for the low densities in the Avignon site is mainly attributed to the bias in the estimation of the number of plants per object due to the classification problem already outlined.

## Discussion and Conclusion

The method proposed in this study relies on the ability to identify plants or group of plants from RGB images. Image classification is a thus a critical step driving the accuracy of the plant density estimation. Wheat plants at emergence have a relatively simple structure and color. The image quality is obviously very important, including the optimal spatial resolution that should be better than 0.4 mm as advised by Jin et al. (2016). Further, the image quality should not be compromised by undesirable effects due to image compression algorithms. As a consequence, when the resolution is between 0.2 and 0.5 mm, it would be preferable to record images in raw format to preserve its quality. A known and fixed white balance should be applied to make the series of images comparable in terms of color. Finally, the view direction was chosen to increase the plant cross section by taking images inclined at around 45° zenith angle in a compass direction perpendicular to the row orientation. Note that too inclined views may result in large overlap of plants from adjacent rows which will pose problems for row (and plant) identification.

Plants were separated from the background based on their green color. A unique unsupervised method based on the Lab transform on which automatic thresholding is applied was used with success across a range of illumination conditions. However, the method should be tested under a much larger range of illumination and soil conditions before ensuring that it is actually applicable in all scenarios. Additionally, attention should be paid to weeds that are generally green. Fortunately, weeds were well-controlled in our experiments. Although this is also generally observed during emergence, weed detection algorithm could be integrated in the pipeline in case of significant infestation. Weeds may be identified by their position relative to the row (Woebbecke et al., 1995). However, for the particular observational configuration proposed (45° perpendicular to the row), the application of these simple algorithms are likely to fail. Additional (vertical) images should be taken, or more refined methods based on the color (Gée et al., 2008) or shape (Swain et al., 2011) should be implemented.

Once the binary images are computed from the original RGB ones, objects containing uncertain number of plants can be easily identified. An ANN method was used in this study to estimate the number of plants from the 13 features of each object. Alternative machine learning techniques were tested including random forest (Breiman, 2001), multilinear regression (Tabachnick et al., 2001) and generalized linear model (Lopatin et al., 2016). The ANN was demonstrated to perform better for the three sites (results not presented in this study for the sake of brevity). The RFE algorithm used to select the minimum subset of features to best estimate the number of plants per object (Granitto et al., 2006) resulted in 4–10 features depending on the data set considered. The features selected are mainly related to the object area and the length of the corresponding skeleton. Conversely, object orientation and extent appear to contribute marginally to the estimation of the number of plants per object. The RFE framework employed here partly accounts for the strong co-dependency between the 13 features considered. The selection process could probably be improved using a recursive scheme similar to the one employed in stepwise regression, or a transformation of the space of the input features.

The wheat population density was estimated with an average of 12% relative error. The error increases with the population density because of the increase of overlap between plants creating larger objects, hence making it more difficult to associate accurately the number of plants they contain. Likewise, a degradation of the performances is also expected when plants are well-developed. Jin and Tang (2009) found that the selection of the optimal growth stage is critical to get accurate estimation of the plant density in maize crops. A timely observation just between Haun stage 1.5–2 corresponding to 1.5–2 phyllochron after emergence appears optimal: plants are enough developed to be well-identified while the overlap between plants is minimized because of the low number of leaves (between 1 and 2) and their relatively erect orientation. However, in case of heterogeneous emergence, it is frequent to observe a delay of about 1 phyllochron (Jamieson et al., 2008; Hokmalipour, 2011) between the first and the last plant emerged. Observation between Haun stage 1.5 and 2 can thus ensure that the majority has emerged. Since the phyllochron varies between 63 and 150°C⋅d (McMaster and Wilhem, 1995), the optimal time window of 0.5 phyllochron (between Haun stage 1.5 and 2) can last about 4–8 days under an average 10°C air temperature. This short optimal time window for acquiring the images is thus a strong constraint when operationally deploying the proposed method.

The success of the method relies heavily on the estimation of the number of plants per object. The machine learning technique used in this study was trained independently for each site. This provides the best performances because it takes into account the actual variability of single plant structure that depends on its development stage at the time of observation, on the genotypic variability as well as on possible influence of the environmental conditions, especially wind. Operational deployment of the method therefore requires the model to be re-calibrated over each new experimental site. However, a single training encompassing all the possible situations may be envisioned in near future. This requires a large enough training data set representing the variability of genotypes, development stage and environmental conditions. This single training data base could also include other cereal crop species similar to wheat at emergence such as barley, triticale, or oat.

Several vectors could be used to take the RGB images, depending mostly on the size of the experiment and the resources available. A monopod and a light rolling platform, the Phenotypette, were used in our study. More sophisticated vectors with higher throughput could be envisioned in the next step, based either on a semi-automatic (Comar et al., 2012) or fully automatic rover (de Solan et al., 2015) or on a UAV platform as recently demonstrated by Jin et al. (2016).

## Author Contributions

The experiment and algorithm development were mainly accomplished by SL and FB. SL wrote the manuscript and FB made very significant revisions. BA also read and improved the final manuscript. All authors participated the discussion of experiment design. BA, PB, and MH significantly contributed to the field experiment in Paris, Toulouse, and Avignon, respectively.

## Conflict of Interest Statement

The authors declare that the research was conducted in the absence of any commercial or financial relationships that could be construed as a potential conflict of interest.
